# Case Report: Cerebral leukodystrophy and the gonadal endocrinopathy: a rare but real association

**DOI:** 10.12688/f1000research.13933.2

**Published:** 2018-04-23

**Authors:** Mohammad Humayun, Abidullah Khan

**Affiliations:** 1 Khyber Teaching Hospital, 25000, Peshawar, Pakistan

**Keywords:** Vanishing white matter, neuro-ovarian failure, leukodystrophy

## Abstract

A 30 year old married Pakistani woman presented in January 2018 with an eight month history of progressive left sided weakness, ataxia, spasticity, underdeveloped secondary sexual characteristics and primary infertility. She was the elder sister of a 19 year old bed bound woman who was diagnosed with vanishing white matter (VWM) disease 12 months previously. The MRI scan of the brain  demonstrated diffuse leukodystrophy and her hormonal assays were significant for premature ovarian insufficiency. Results from her genetic tests demonstrated a point mutation in eukaryotic initiation factor 2B (EIF2B). Thus, she was the second confirmed case of  VWM from her  family of 12 siblings with normal parents.

## Introduction

Van der Knaap described a new clinical entity of neuro-ovarian failure in 1996 as vanishing white matter(VWM) disease ^1^. This rare entity is also known as ovarioleukodystrophy and is caused by a mutation of the eukaryotic initiation factor 2B (EIF2B) ^2^. It most commonly presents in infants or early childhood as a progressive central neuronal failure causing limb weakness, spasticity, cognitive decline, seizures, encephalopathy and ultimately death ^2, 3^. The onset in adults is very rare and in female patients, is frequently associated with premature ovarian failure ^3^. In March 2017, we reported the first ever case of VWM affecting a 19 year old woman from Pakistan ^4^. Herein, we report the case history of her elder sister diagnosed as VWM in January 2018.

## Case report

A 30 year old Pakistani woman, presented in January 2018 with an eight month history of progressive left side weakness in pyramidal distribution. She had developed spasticity in the left lower limb and had partial contractures affecting both of her hands. Since the previous month, she felt clumsy in her right arm and leg. She had cerebellar signs and bilateral optic atrophy. However, the rest of her cranial nerves, the spine and the sensory system were normal. She was married for the last four years and had never conceived. Her menstrual cycles began at the age of 17 years. Her initially normal four-weekly cycles became more irregular five years down her menarche leading first to oligomenorrhea and then secondary amenorrhea at the age of 25 years. She had underdeveloped secondary sexual characteristics. Her mental state examination was normal. There was no precedent history of any trauma, surgery, malignancy or infection.

**Figure 1.**
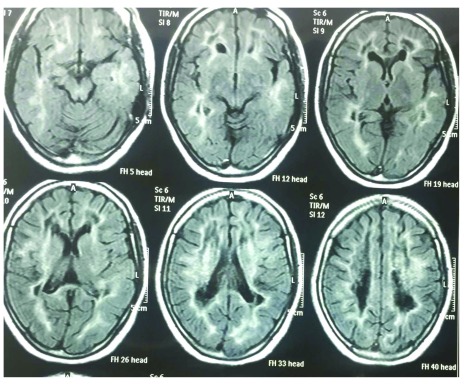
Cross-sectional view of T-2 weighted imaging with FLAIR demonstrating loss of cerebral white matter.

**Figure 2.**
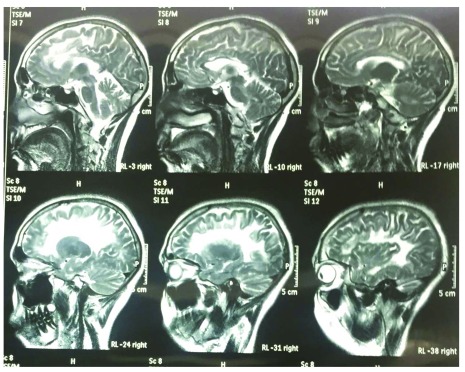
Saggital view of T-2 weighted images of the brain without FLAIR.

Her MRI brain scan was remarkable for diffuse leukodystrophy ( Figure 1 and Figure 2). Her hormonal assays were consistent with premature ovarian insufficiency ( Table 1). An ultrasound of her abdomen and pelvis was remarkable for small ovaries and uterus. Her lumbar puncture results were normal and there were no oligoclonal bands in her cerebrospinal fluid (CSF). She underwent genetic tests including sequence analysis and polymerase chain reaction (PCR), which demonstrated a point mutation in the EIF2B4 gene. Thus, she was diagnosed as a second case of VWM from the same family of 12 siblings and 2 parents. None of the family members were screened due to affordability issues.

**Table 1.** Laboratory results for Hormonal Assays.

Test	Results	Normal Range
Follicular Stimulating Hormone (FSH)	143 mIU/ml	1.5–12.4 mIU/ml
Luteinizing Hormone (LH)	57.21 mIU/ml	1.7–8.6 mIU/ml
Total Estrogen	5 pg/ml	50–300 pg/ml

She was counseled and vaccinated against the common pathogens. She was prescribed baclofen 20mg/day and clonazepam 0.5mg/day for her spasticity. She will be reviewed again in 6 months time. The follow-up plan includes a detailed physical assessment and a repeat MRI brain scan.

## Discussion

Ovarioleukodysptrophy or vanishing white matter disease (VWM) is a rare autosomal recessive disorder. It has central neuronal presentation in the form of progressive ataxia, spasticity, and variable optic atrophy in combination with endocrinopathy manifesting as premature ovarian failure ^3, 4^. This disease has protean spectrums of presentation ranging from the most severe prenatal and infantile forms to the relatively less severe adult onset varieties ^5^. Our second patient had the adult onset disease, a phenotype similar to her younger sister.

The mildest of all, the adult onset variant of VWM presents with a combination of neurological features including pyramidal weakness, cerebellar signs and optic atrophy in association with premature ovarian failure ^4, 5^. The sensory system, the cognitive function and the rest of the cranial nerves are relatively spared, at-least initially ^4^. Our patient presented with limb weakness, ataxia and visual loss in association with primary infertility due to ovarian failure. Her presentation was different from her younger sister in that, she did not have seizure or dementia ^4^.

The diagnostic workup includes MR imaging of the central nervous system which demonstrates diffuse and cystic degenerative loss of the deep cortical white matter and U-fibers. The grey matter is preserved ^3, 5, 6^. This is due to a mutation in EIF2B which causes impairment of protein synthesis under conditions of cellular stress like infection, trauma, intense emotions and surgery. The genetic tests confirm the diagnosis ^6^. Those females who live into adulthood develop ovarian failure ^2, 4, 6^. Interestingly, according to our literature search, primary testicular failure has not been described in any reported case of affected males.

Treatment is largely palliative and preventive. This may include avoidance of stressors, vaccination, anti epileptic drugs and hormonal replacement therapy in affected females ^4, 5, 7^.

## Conclusions

VWM is a rare but potentially serious disease. In adult females, premature ovarian insufficiency is characteristic. There is no effective treatment.

## Consent

Written informed consent for publication of her clinical details and/or clinical images was obtained from the patient.

## Data availability

All data underlying the results are available as part of the article and no additional source data are required.
