# Supplementary material for: Case Report: Cerebral leukodystrophy and the gonadal endocrinopathy: a rare but real association
Source: F1000Res. 2018 Apr 23;7:158. Originally published 2018 Feb 7. [Version 2] doi: 10.12688/f1000research.13933.2 (PMC5915757; doi:10.12688/f1000research.13933.2)
Supplement: Peer review history

# Peer review: Referee response for version 2

**DOI:** 10.5256/f1000research.15905.r33345

**Published:** 2018-04-24

**Authors:** Bernard Corenblum

**Affiliations:** 1 Division of Endocrinology & Metabolism, Department of Medicine, University of Calgary, Calgary, AB, Canada

My minor concerns were adequately addressed.

 I have no concerns with the revised edition, and recommend it being accepted.

 I personally did learn from this report.

I have read this submission. I believe that I have an appropriate level of expertise to confirm that it is of an acceptable scientific standard.

# Peer review: Referee response for version 1

**DOI:** 10.5256/f1000research.15146.r32796

**Published:** 2018-04-12

**Authors:** Bernard Corenblum

**Affiliations:** 1 Division of Endocrinology & Metabolism, Department of Medicine, University of Calgary, Calgary, AB, Canada

Premature menopause is a term no longer accepted. Premature ovarian insufficiency is used now.

 The endocrine aspect of this case is the hypogonadism due to ovarian failure. No speculation is given as to the association, but perhaps none is known.

 She presented at age 30, older than most, including her sister, yet was sexually immature in development.

 There should be more detail on the reproductive history: onset of menarche, cycle history, when did this change, etc. This phenotype appears to be a spectrum with this case later in onset than usual, so more details are needed to extend the data base on this clinical association.

 Is there any relationship with adrenal insufficiency?

I have read this submission. I believe that I have an appropriate level of expertise to confirm that it is of an acceptable scientific standard, however I have significant reservations, as outlined above.

# Response

**Published:** 2018-04-15

**Authors:** Abidullah Khan

**Affiliations:** Warrington and Halton Hospitals, NHS, UK

We thank Dr.Bernard Corenblum for his precious time in reviewing our manuscript. The points raised by him are praise worthy and we did our best to address his concerns. The points addressed in the light of his recommendation will definitely improve the quality of our article.

1. The term 'premature menopause' has been rephrased as 'premature ovarian insufficiency'.
2. More details have been provided regarding her reproductive history including onset of menarche, cycle history and the time they changed. 
3. There is no association with adrenal failure. Association of adrenal failure with leukodystrophy is a second well defined entity called as 'adreno-leukodystrophy'.

Thanks again.

# Peer review: Referee response for version 1

**DOI:** 10.5256/f1000research.15146.r30650

**Published:** 2018-03-22

**Authors:** Jeremy Chataway

**Affiliations:** 1 Queen Square Multiple Sclerosis Centre, Institute of Neurology, University College London, London, UK

This report look at Vanishing White Matter disease (VMD), part of the genetically inherited leukoencephalopathies, a rare group of neurological disorders, of which X-linked adrenoleukodystrophy is the most common (1/17,000). VMD is an autosomal recessive disorder characterised by progressive neurological impairment and white matter cystic degeneration. It generally presents in childhood but adult cases are well reported.

 Here a 30 year old woman reports a short duration of weakness, ataxia and primary infertility. An older sister also has VMD (there are siblings in total).

 In females there can be ovarian failure, which is a useful diagnostic feature, as is shown here (so it can be described as an ovarioleukodystrophy). The periventricular white matter shows the same signal intensity as CSF on FLAIR/T2 weighted imaging. Mutations occur in any of the 5 genes that code for translation initiation factor EIF2B ( *EIF2B1 – EIF2B5*), here is EIF2B. Sometimes deterioration is triggered by physical or emotional trauma.

 This is a useful addition to the literature of a very rare group of conditions.

I have read this submission. I believe that I have an appropriate level of expertise to confirm that it is of an acceptable scientific standard.

## References

[1] AhmedRMMurphyEDavagnanamIPartonMSchottJMMummeryCJRohrerJDLachmannRHHouldenHFoxNCChatawayJ: A practical approach to diagnosing adult onset leukodystrophies. *J Neurol Neurosurg Psychiatry*.2014;85(7) : 10.1136/jnnp-2013-305888 770-81 10.1136/jnnp-2013-305888 24357685
